# A "Revolt" of Sanatorium Patients

**Published:** 1915-11-20

**Authors:** 


### A "Revolt" of Sanatorium Patients.

It is some time since we have had to report an outburst against discipline on the part of sanatorium patients, but that these regrettable occurrences still arise is seen by an incident that happened last Sunday week at the South Staffordshire Joint Tuberculosis Sanatorium at Moxley, near Birmingham. Apparently it has been the rule of the institution for patients to take a walk on Sunday mornings between 9.30 and 11.15. On the Sunday in question the patients, it appears, were informed that their customary walk was to be within the grounds of the institution, whereupon sixteen, about two-thirds of the total number, refused to obey and went outside for a walk. On their return at the usual hour they were summoned, according to accounts, into a room in the building, where three of their number were singled out and told to leave the sanatorium since they would not obey its rules. The remainder, however, out of sympathy or by way of protest, immediately decided also to leave, whereupon the sixteen left the institution, notwithstanding the difficulties presented by a Sunday service of trains. Manifestly the rights and wrongs of the dispute are contained not in such published statements as those which the departing patients have hastened to furnish, but in the unstated cause for the order forbidding the customary walk. Likely enough previous misconduct, real or imagined, led to the abrogation of the privilege, but, till this fact be known, the authorities must not be condemned, though the good administrator is one who does not allow matters to develop into "a crisis" of this character without the gravest reason. We understand that the medical officer in charge of the sanatorium has been lately appointed, and since writing the above we learn that two of the patients have returned, and, on apology, have been readmitted. The practice of breaking bounds is difficult to combat, and two or three evilly disposed ringleaders can induce breaches of discipline.

